# Environmental Fate of Double-Stranded RNA in Agricultural Soils

**DOI:** 10.1371/journal.pone.0093155

**Published:** 2014-03-27

**Authors:** Samuel Dubelman, Joshua Fischer, Fatima Zapata, Kristin Huizinga, Changjian Jiang, Joshua Uffman, Steven Levine, David Carson

**Affiliations:** Regulatory Division, Monsanto Company, St. Louis, Missouri, United States of America; Ghent University, Belgium

## Abstract

A laboratory soil degradation study was conducted to determine the biodegradation potential of a DvSnf7 dsRNA transcript derived from a Monsanto genetically modified (GM) maize product that confers resistance to corn rootworm (CRW; *Diabrotica spp*.). This study provides new information to improve the environmental assessment of dsRNAs that become pesticidal through an RNAi process. Three agricultural soils differing in their physicochemical characteristics were obtained from the U.S., Illinois (IL; silt loam), Missouri (MO; loamy sand) and North Dakota (ND; clay loam), and exposed to the target dsRNA by incorporating insect-protected maize biomass and purified (*in vitro*-transcribed) DvSnf7 RNA into soil. The GM and control (non-GM maize) materials were added to each soil and incubated at ca. 22°C for 48 hours (h). Samples were collected at 12 time intervals during the incubation period, extracted, and analyzed using QuantiGene molecular analysis and insect bioassay methods. The DT_50_ (half-life) values for DvSnf7 RNA in IL, MO, and ND soils were 19, 28, and 15 h based on QuantiGene, and 18, 29, and 14 h based on insect bioassay, respectively. Furthermore, the DT_90_ (time to 90% degradation) values for DvSnf7 RNA in all three soils were <35 h. These results indicate that DvSnf7 RNA was degraded and biological activity was undetectable within approximately 2 days after application to soil, regardless of texture, pH, clay content and other soil differences. Furthermore, soil-incorporated DvSnf7 RNA was non-detectable in soil after 48 h, as measured by QuantiGene, at levels ranging more than two orders of magnitude (0.3, 1.5, 7.5 and 37.5 µg RNA/g soil). Results from this study indicate that the DvSnf7 dsRNA is unlikely to persist or accumulate in the environment. Furthermore, the rapid degradation of DvSnf7 dsRNA provides a basis to define relevant exposure scenarios for future RNA-based agricultural products.

## Introduction

Genetically modified (GM) crops expressing traits that confer protection from insect pests and facilitate weed management provide significant value in agriculture and require novel modes of action for sustainable efficacy. Comprehensive science-based ecological risk assessments (ERA) are required to ensure that products with benefits for agriculture are also safe for the environment. Ecological risk from the cultivation of a GM crop will be a function of both potential harm (hazard) and environmental exposure [1]. Determining the fate of a transgenic trait in soil, whether it be an insecticidal protein or dsRNA, is a component of the ERA for a GM crop, and defines realistic routes and levels of exposure to non-target organisms in the environment.

With the introduction of GM crops, attention has focused on the environmental fate of transgenic-derived DNA and proteins. The environmental exposure of Cry proteins derived from *Bacillus thuringiensis (Bt)* is minimal due to the lack of persistence of *Bt* proteins in soil [2], [3]. For example, laboratory studies measuring the degradation rate of the Cry1Ac protein expressed in *Bt* cotton showed that Cry1Ac has a half-life (DT_50_) of 16 days (d) when incorporated into soil microcosms [4]. Comparable results were reported for Cry1Ab from laboratory microcosm studies with *Bt* maize [5], [6]. Results obtained from laboratory soil degradation studies were confirmed in multi-year monitoring studies of large agricultural fields with continuous cultivation of the *Bt* crops, which showed no detection, persistence, or accumulation of Cry1Ac in *Bt*-cotton fields [7] or of Cry1Ab in *Bt*-maize fields [8], [9]. Based on the results of these laboratory and field studies, EPA has concluded that *Bt* proteins do not accumulate as a result of continuous cultivation [3].

Similar to the case of *Bt* proteins, the rate of nucleic acid degradation can be influenced by the biophysical characteristics of soil and sediments in addition to other abiotic and biotic factors [10]–[13]. DNA degradation kinetics have been defined in several instances using plant tissue or extracellular DNA applied to microbiologically active soil and sediment. For example, genomic DNA (gDNA) from tomato leaves exhibits a half-life of about 1.5 d when introduced to viable soil [14]. Similarly, transgenic and conventional soybean DNA from freeze-dried leaf tissue exhibits a half-life of about 1.4 d [15]. Once DNA is released from plant tissues or applied directly to soil, more rapid and extensive degradation occurs. Purified GM and non-GM maize DNA exposed to leachate water at ambient room temperature exhibits a half-life of <2 h while GM and non-GM soybean DNA exposed to similar conditions has a half-life of about 4 h [16].

Degradation of DNA in the soil environment is likely due to processing by DNases. High molecular weight gDNA molecules may first be cleaved into oligonucleotides of approximately 400-bp by microbial restriction endonuclease I activity. These oligonucleotide substrates can then undergo further degradation through the activity of microbial-produced DNases. Bacterial populations may also increase by approximately one order of magnitude in soil exhibiting DNA-degrading activity [17].

Less information is available on the degradation kinetics of RNA in soil, however data collected to date provides no evidence that RNA persists any longer than DNA [18], [19]. In Keown *et al.*, soil samples amended with either dried DNA or RNA were analyzed for released nitrogen and CO_2_ evolution as a measure of nucleic acid degradation. The results of this study found that RNA degraded as quickly as DNA in different soils [19].

Crops that express double-stranded RNA (dsRNA) molecules are being developed which take advantage of the endogenous RNAi machinery of target insects and can produce highly specific insecticidal oligonucleotides (siRNA) for agricultural pest control. DvSnf7 dsRNA expressed in GM maize confers protection against the Western Corn Rootworm (WCR) [20]. Ingestion of DvSnf7-expressing tissue by a susceptible insect host results in the uptake of the dsRNA leading to mRNA and protein suppression of a targeted essential gene in WCR, systemic spread, and eventual mortality [20], [21]. Because of the potential for increased development of products with RNA-based modes of action, more information is needed to understand the fate of dsRNA in the soil environment [22], [23]. Results reported here assessing the environmental fate of DvSnf7 dsRNA from an insect-protected maize product indicate that the insecticidal dsRNA degrades readily and is unlikely to persist in the soil environment.

## Materials and Methods

### Synthesis of IVT DvSnf7

*In vitro* T7 RNA Polymerase-transcribed 968 nucleotide (nt) DvSnf7 RNA (IVT DvSnf7 RNA) was used to treat soil samples and serve as the reference standard for the QuantiGene 2.0 microplate assay [24]. This 968-nt transcript is produced in transgenic maize containing a DvSnf7 suppression cassette and was determined by cDNA sequencing to include a 240-nt inverted repeat region and adjacent 5′- and 3′ sequences. The 968-nt DvSnf7 fragment was amplified by PCR from the original plant transformation vector and cloned downstream of a synthetic T7 promoter sequence (TAATACGACTCACTATAGGG) in pUC19 plasmid. The identity of the recombinant pUC plasmid template used to produce the IVT DvSnf7 RNA was verified by DNA sequencing. For IVT RNA synthesis, the pUC plasmid was linearized by BglII restriction digestion and incubated with nucleoside triphosphates (8 mM each, Sigma) and T7 RNA Polymerase in transcription buffer overnight at 37°C. The reaction was then treated with DNase I (100 units per 1 mL, Ambion) and extracted with phenol∶chloroform (1∶1 volume∶volume). The size of the IVT DvSnf7 RNA was confirmed by agarose gel electrophoresis and its concentration was determined using a NanoDrop 8000 (Thermo Scientific, Wilmington, DE) according to the manufacturer's instruction. Aliquots of the IVT DvSnf7 RNA sample were stored in a −80°C freezer.

### Soil Sample Preparation and Dosing

Three soil types were collected from the top 15 cm of fields in Illinois (IL), Missouri (MO) and North Dakota (ND) and subsequently characterized for percent sand, silt and clay; USDA textural class; bulk density; percent organic matter; pH; cation exchange capacity (CEC); field moisture capacity (FMC) at 1/3 Bar and at 15 Bar; and a number of chemical elements (AGVISE Laboratories, Northwoods, ND). The three soils were sieved at AGVISE Laboratories through a 2 mm screen to remove non-soil debris prior to analysis. Upon receipt, each soil was spread on large trays to air dry and then repacked and stored at ambient temperature under aerobic conditions. At the start of the study, 1 g soil samples were weighed into 50 ml conical tubes. Samples containing maize tissue were amended with a mixture of DvSnf7-expressing lyophilized root and shoot tissue (20∶80 w/w) derived from V8-V10 growth stage plants at a rate of 20 mg tissue per g soil. Due to relatively low expression of DvSnf7 in maize tissue, a solution of IVT DvSnf7 was diluted in 1× PBST (1 mM KH_2_PO_4_, 10 mM Na_2_HPO_4_, 137 mM NaCl, 2.7 mM KCl, pH 7.0; Roche Diagnostics, Indianapolis, IN) to the desired concentrations. Given the mass ratio of plant tissue to soil in the samples, the QuantiGene and insect bioassay methods were not sufficiently sensitive to generate quantifiable measurements of the endogenously expressed DvSnf7 RNA over the entire experimental range (90% degradation). Samples were therefore treated with an IVT DvSnf7 RNA solution of sufficient concentration to detect dsRNA after 90% of the initial DvSnf7 RNA had degraded. The IVT DvSnf7 solution was diluted such that ∼400 µl solution was required for dosing each sample. Samples were incubated at ambient temperature for the required interval and then placed in −80°C storage until all samples were collected and prepared for extraction.

### Sample Extraction

All samples were extracted prior to QuantiGene analysis and insect bioassay. Briefly, 25 steel ball bearings were added to each sample in 50 ml conical tubes. For Illinois and Missouri soil samples, sufficient 1× PBST pH 7 was added to achieve a 25∶1 buffer∶soil ratio. For high clay soil samples (e.g. ND), the pH 7 buffer was substituted with 1× PBST pH 12 to improve the extraction efficiency of clay-bound RNA. Samples were then agitated in a paint shaker (Harbil), for 10 minutes and passed through a 0.22 µm vacuum filter. Samples containing high clay ND soil were centrifuged for 10 minutes at 7500× *g* prior to filtration to avoid clogging the filter with fine clay particles. Filtered sample extracts were aliquoted and frozen at −80°C until analyses were performed.

### QuantiGene Analysis

Prior to sample analysis by a DvSnf7-specific QuantiGene assay (Affymetrix, Inc., Santa Clara, CA), aliquots of sample extracts were purified to remove potential hybridization inhibitors (e.g. humic acid, polyphenols). Briefly, 1 ml aliquots of the sample extracts were mixed with an equal volume of citrate-buffered phenol pH 4.3 (Sigma-Aldrich, Saint Louis, MO) and chloroform (Sigma-Aldrich, Saint Louis, MO). Emulsified samples were separated via centrifugation and the RNA was precipitated from the aqueous phase with isopropanol. Following precipitation, samples were centrifuged, air-dried, and resuspended in DEPC-treated water (Sigma-Aldrich, Saint Louis, MO). The concentration of DvSnf7 RNA in purified samples was determined with a QuantiGene 2.0 assay following the manufacturer's instructions. A custom QuantiGene probe set was designed by the manufacturer to hybridize to the sequence within DvSnf7 and IVT DvSnf7 RNA that confers insecticidal activity. The IVT DvSnf7 RNA and the sequence-specific recognition by the QuantiGene probe set has previously been described in detail [24]. Each purified sample was diluted 1∶100 in assay diluent and analyzed in triplicate. The DvSnf7 concentration was calculated using a four-parameter curve fit [25] and the mean concentration of each sample was calculated from triplicate analysis.

### Determination of DvSnf7 RNA Concentration by SCR Mortality Assay

Southern Corn Rootworm (SCR) was selected for these bioassays because it was more amenable than Western Corn Rootworm (WCR) to laboratory bioassays and displayed comparable sensitivity to the DvSnf7 RNA [20]. Four separate soil samples were collected at each of 12 collection times for each of the three soil types and extracted prior to assay. Three replicates of each of these extracts were used for insect bioassays. Each bioassay determination included a concentration response curve with several sequential dilutions of *IVT* DvSnf7 RNA that served as a standard curve to quantify DvSNf7 RNA in soil extracts. For each bioassay, extract solutions were prepared by aliquoting each test and control soil sample extract into three replicates, diluting with purified water, incorporating the dilution into an agar-based SCR diet (Bio-Serv, #F9800B) and then vortex-mixing the solution until visually homogeneous. These diet mixtures were then dispensed in 0.25 ml aliquots into 48-well plates (Becton Dickson Labware, Franklin Lakes, NJ; Prod. # 3078). SCR larvae (≤30 h after the first observation of hatching) were individually placed on these diets with a target number of 24 insects tested for each sample replicate. The insects were incubated for a period of 12 d at 27°C, 70% relative humidity and in total darkness. Assays were evaluated for survival at the end of the incubation period and results were subjected to a correction to account for control mortality. Standard curves for SCR bioassays were analyzed by probit analysis under PROC PROBIT in SAS, the OPTC function was used to correct for control mortality, and DvSnf7 soil concentrations were estimated by inverse prediction.

### Environmental Fate Modeling

A standard 3-parameter logistic equation [26], run under PROC NLMIXED within SAS (Release 9.3), was used to model the time-dependent decrease in DvSNf7 RNA concentration in soil for the QuantiGene and insect bioassay data, and to estimate DT_50_ and DT_90_ values, the time periods required for 50% and 90% degradation of the initial DvSnf7 RNA concentration in soil.

## Results

The environmental fate of DvSnf7 dsRNA was determined in three different soils that have physical characteristics representative of world regions where maize is grown, including a silt loam (IL), loamy sand (MO), and clay loam (ND) soil. Prior to the study, the three soils were characterized by determining percent sand, silt and clay, USDA textural class, bulk density, percent organic matter, pH, cation exchange capacity (CEC), and field moisture capacity (FMC) at 1/3 Bar and 15 Bar (Table 1).

**Table 1:** Physicochemical characteristics of three soils used to determine the environmental fate of DvSnf7 RNA.

	Soil Characterization Results		
Parameter	Illinois	Missouri	North Dakota
USDA Textural Class:	Silt Loam	Loamy Sand	Clay Loam
Particle Size Distribution			
% Sand	23	86	26
% Silt	60	11	38
% Clay	17	3	36
Bulk Density (g/cm^3^)	0.92	1.3	0.92
% Organic Matter	3.5	1.7	5.1
pH	5.8	5.5	7.1
Cation Exchange Capacity (meq/100 g)	8.5	6.0	24.4
Field Moisture Capacity (ml/100 g) at 1/3 Bar	28.9	6.9	37.1
at 15 Bar	9.6	3.6	25.1

**Figure 1:**
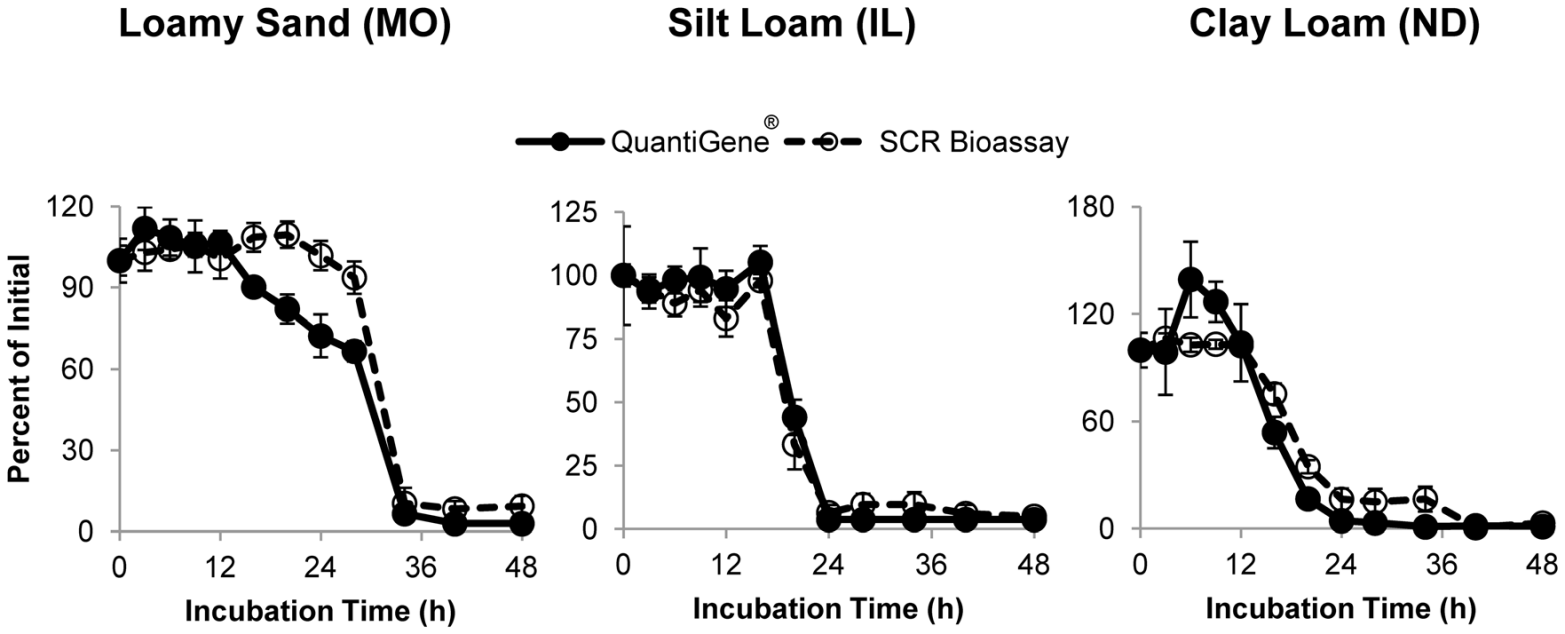
DvSnf7 RNA degrades rapidly when applied to soil. Soil samples were amended with lyophilized maize tissue and fortified with 7.5 µg DvSnf7 RNA per gram of soil. DvSnf7 RNA concentration in soil was measured with a QuantiGene assay. Biological activity against Southern Corn Rootworm was determined by assessing insect mortality in a 12-day bioassay. Error bars represent one standard error of the mean (n = 4).

Because DvSnf7 dsRNA is expressed in GM maize, soil samples were amended with a mixture of lyophilized root and shoot maize tissue to simulate post-harvest field environmental conditions. Given that the *in planta* expression of DvSnf7 RNA is low, tissue-amended soil samples were also supplemented with a solution of IVT DvSnf7 dsRNA to ensure that a robust quantitation could be obtained at all time intervals of an experimental range covering at least 90% degradation of the initial DvSnf7 RNA concentration. The concentration of DvSnf7 in soil samples collected at 12 time intervals during the 48 h incubation was determined using a QuantiGene assay. The QuantiGene assay detects both *in planta*-expressed DvSnf7 and *in vitro*-transcribed DvSnf7 via hybridization with target sequence-specific probes. After an initial induction period of a few hours, DvSnf7 RNA degraded rapidly in all three soils and was no longer detected by QuantiGene after 24–36 h (Figure 1). As a control, a solution of DvSnf7 incubated at ambient temperature in the absence of soil or maize tissue was stable after 48 h and showed no signs of degradation (data not shown). The biological activity of DvSnf7 RNA in these samples was also measured by a mortality bioassay using Southern Corn Rootworm (SCR) as a sensitive target species. It has previously been reported that the dsRNA produces an insect mortality response when the double stranded segments are ≥60 base pairs [20] and this result is consistent with the findings of Miller *et al.* [27]. Results indicate that the biological activity (insect mortality) of DvSnf7 RNA declined rapidly in all three soils similar to results obtained using the QuantiGene assay. While the QuantiGene assay has the potential to detect partially degraded dsRNA species in addition to the parent compound (see reference 24), the high degree of correlation observed between the QuantiGene and the insect-bioassay results suggests that the dsRNA is being degraded to less than 60 bp at minimum. Insect bioassay results exhibited a similar initial induction period, followed by a rapid degradation phase, and ultimately declining to a level indistinguishable from the background mortality of the control insect group (Figure 1).

Degradation kinetics of DvSnf7 RNA in soil amended with maize tissue were calculated using a 3-parameter logistic equation. The estimated DT_50_ values ranged from about 14 h (ND soil) to about 29 h (MO soil) using results obtained from QuantiGene and SCR bioassay (Table 2). The estimated DT_90_ values ranged from 21 to 35 h for the three different soils. Furthermore, these results demonstrated high consistency between two analytical methods that rely on uniquely different end-points (molecular hybridization versus insect mortality). These results indicate that the DvSnf7 RNA was degraded and devoid of any detectable biological activity within 2 days of incorporation into soil.

**Table 2:** QuantiGene and insect bioassay methods provide comparable degradation rate estimates.

		QuantiGene			Insect Mortality		
Soil	Rate Estimate	Time (±SE)	LCI	UCI	Time (±SE)	LCI	UCI
Loamy Sand (MO)	DT_50_	27.8 (0.6)	26.6	29.1	29.0 (1.2)	26.4	31.5
	DT_90_	34.5 (0.5)	33.4	35.6	34.3 (0.8)	32.5	36.1
Silt Loam (IL)	DT_50_	19.4 (0.4)	18.6	20.3	17.7 (1.4)	14.6	20.9
	DT_90_	23.0 (0.3)	22.3	23.6	23.1 (1.1)	20.5	25.6
Clay Loam (ND)	DT_50_	15.1 (0.7)	13.7	16.4	13.7 (0.8)	11.9	15.6
	DT_90_	21.6 (0.6)	20.5	22.7	20.5 (0.8)	18.8	22.2

The degradation rate estimates (DT_50_ and DT_90_), in hours, were calculated for DvSnf7 RNA in each soil type as measured by QuantiGene and insect bioassay. Numbers in parenthesis represent the standard error of the mean (SE) in hours. LCI and UCI represent the lower and upper 95% confidence intervals respectively, in hours.

**Figure 2:**
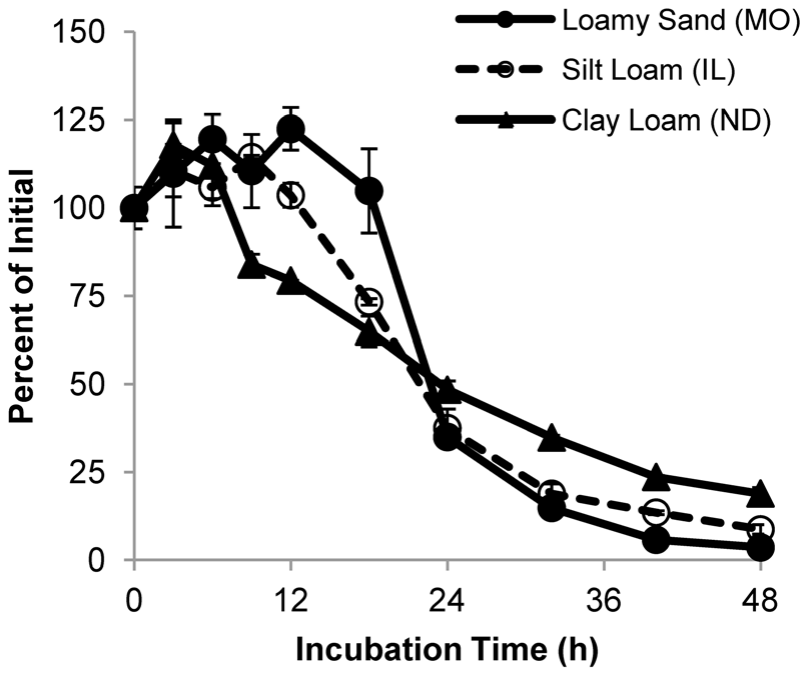
DvSnf7 RNA degrades in soil alone. Soil samples were fortified with 7.5 µg DvSnf7 RNA per gram of soil in the absence of maize tissue. DvSnf7 RNA concentration was determined with a QuantiGene assay. Error bars represent one standard error of the mean (n = 2).

Since DvSnf7 RNA was observed to degrade rapidly in soils amended with maize tissue, we tested whether dsRNA would be degraded with similar kinetics when applied to soil alone. The three representative soils were treated with a solution of IVT DvSnf7 dsRNA and incubated at ambient temperature for up to 48 h. Analysis by QuantiGene demonstrated that regardless of soil class, DvSnf7 RNA degraded rapidly in soil alone, with similar kinetics to soil amended with maize tissue (Figure 2). These results indicate that dsRNA degrades rapidly in soil, in the presence or absence of maize biomass.

**Figure 3:**
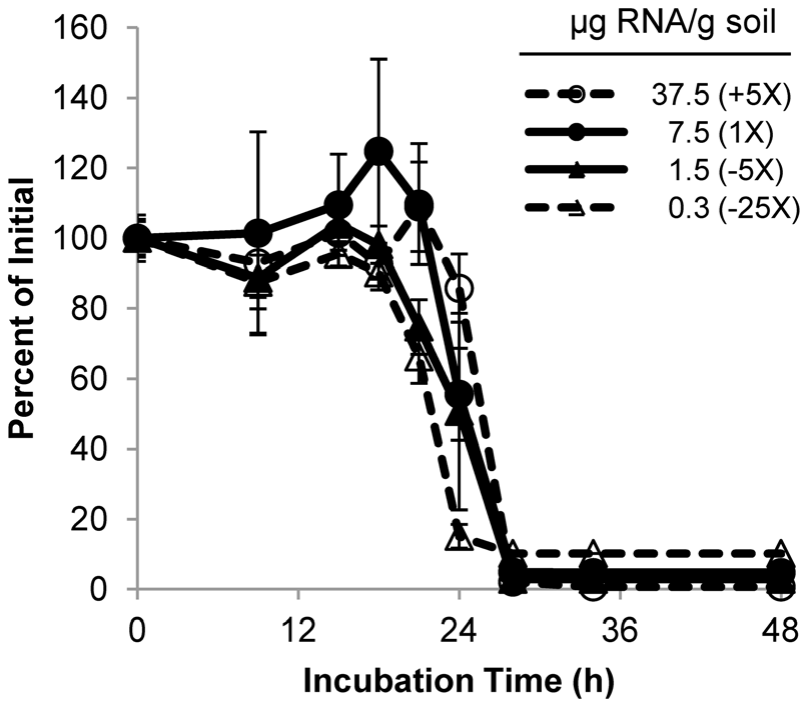
Degradation kinetics for DvSnf7 RNA are independent of initial dsRNA concentration. Soil samples were amended with lyophilized maize tissue and fortified with the indicated amounts of DvSnf7 RNA (µg RNA/g soil). Soil concentration of DvSnf7 RNA was determined with a QuantiGene assay. Numbers in parenthesis indicate the relative DvSnf7 RNA treatment. Error bars represent one standard error of the mean (n = 3).

To determine whether the observed DvSnf7 degradation kinetics are dependent on the amount of dsRNA applied to the soil microcosms, the relationship between degradation kinetics and the initial dsRNA concentration was investigated. Tissue-amended silt loam soil (IL) was treated with four different DvSnf7 RNA concentrations ranging from to 0.3 to 37.5 µg per gram of soil and degradation kinetics of the dsRNA was measured at each treatment level. The lowest treatment level (0.3 µg/g) was the lowest initial dsRNA concentration that would allow quantifiable QuantiGene measurements after at least 90% degradation of the applied dsRNA. Despite varying the amount of DvSnf7 added to soil by more than two orders of magnitude, there was no significant observable change in dsRNA degradation kinetics (Figure 3). These results suggest that the rate of dsRNA degradation is largely independent of the dsRNA concentration applied to soil.

## Discussion

This study demonstrates that the DvSnf7 dsRNA rapidly degrades in the soil environment with a half-life of less than 30 h. This degradation is relatively rapid when compared to *Bt* proteins that exhibit half-lives typically ranging from approximately one to several days [4], [5], [8], [28]–[30]. The biodegradation of dsRNA was established using laboratory microcosms containing soil alone or amended with maize tissue to simulate the composition of a maize field following harvest. Biodegradation rate data obtained from laboratory soil microcosms has proven to be a reliable indicator for predicting potential persistence of a given organic substrate in more complex environmental or field conditions [4], [31], [32], and the lack of persistence or accumulation of the Cry1Ab [8], [9] and Cry1Ac [7] proteins has been demonstrated in field dissipation studies. Following an initial lag period, DvSnf7 dsRNA degrades rapidly as measured by quantitative molecular hybridization and by assessing the loss of biological activity (mortality) using a sensitive insect bioassay. The observed lag period (<10 h) is likely due to acclimation of the resident microbial community to the amended maize tissue or dsRNA and is a common feature of organic substrate biodegradation [33].

Viable resident microbial communities have been shown to be necessary for the decomposition of leaf litter and plant genomic DNA [14]. Degradation of the dsRNA in acclimated soil microcosms could result from direct metabolism by the resident microbial community or from plant-encoded nucleases contained within the amended maize tissue [34]. To test this, DvSnf7 RNA was incorporated directly into soil in the absence of maize tissue. Similar to soil microcosms amended with maize tissue, there was an initial acclimation phase followed by rapid biodegradation. The estimated half-life of DvSnf7 RNA was less than 24 h in soil alone regardless of soil type, which indicates that the activity of the resident soil microbial community is sufficient to degrade dsRNA. This finding has particular implications for exposure assessments of potential nucleic acids formulated for applications in which an exposure scenario may not necessarily include the presence of plant detritus. Consistent with our findings for dsRNA, Southern hybridization of phage P22 DNA recovered from soil microcosms indicates that DNA undergoes rapid degradation within 5–24 h of application. Furthermore, an increase in observable counts of soil microorganisms temporally coincides with degradation of the applied DNA. Evidence suggests this process is mediated significantly by the soil bacterial communities as degradation can be impeded by adding rifampicin and chloramphenicol, but not cyclohexamide [17].

The biodegradation of dsRNA is also likely mediated by microbial-produced ribonucleases [12], [17]. Gulden and colleagues demonstrated that the half-life of DNA sequences decreased with increasing temperature implicating enzymatic processes are responsible for degrading free nucleic acid [12], [16]. For RNA, various ribonucleases (RNases) produced by pro- and eukaryotes may play critical roles in its processing and degradation. In particular, RNase III is an endoribonuclease that is responsible for the cleavage of dsRNA substrates [35]. Recently, the conserved Eri1 family of RNases was shown to degrade double-stranded silencing RNAs [36], [37] such as those that may be used in GM agricultural products.

When RNA is released into the environment, the same RNases responsible for intra-cellular degradation are active in an extra-cellular capacity. Single-stranded RNA (ssRNA) from viral genomes exhibit a half-life on the order of days in seawater [38]. Similarly, RNA from human norovirus added to seawater and incubated under simulated winter-like conditions (8±0.5°C and UV radiation at 1 mW/cm^2^) exhibited an exponential decrease in concentration with a DT_90_ of approximately 141 h and no detectable amount of RNA found after 14 d [39]. In sand, free extracellular microbial RNA labeled with ^14^C and ^3^H exhibited up to 62% degradation after 14 d [40].

In comparison to *Bt* proteins, the expression of DvSnf7 *in planta* is relatively low such that addition of a DvSnf7 RNA solution was required to produce a full and quantitative degradation profile in soil microcosms. Therefore, we tested whether the degradation kinetics were dependent on the concentration of dsRNA applied to soil. Despite varying the amount of dsRNA in soil microcosms by over two orders of magnitude, there was little observed change in the half-life of DvSnf7. Furthermore, there was no discernible correlation between the applied amount of the dsRNA and efficiency of analytical recovery from soil suggesting the soil microcosms were not saturated with the applied nucleic acid (data not shown). Consistent with these results, saturation was not observed when DNA was applied to microcosms over a range of concentrations up to 28 µg of DNA per gram of soil [17]. Overall, results of these experiments suggest that the dsRNA expressed *in planta* by this GM maize is unlikely to persist in the soil environment.

In summary, we have demonstrated the environmental degradation of dsRNA in multiple soil environments. The degradation of dsRNA was characterized by an initial acclimation phase followed by a rapid loss of DvSnf7 RNA and its associated biological activity in three different soils, exhibiting half-lives ranging from about 14 to 30 h. Rapid degradation of the dsRNA was observed in soil alone or soil amended with maize tissue and the rate of degradation was largely independent of the RNA treatment level. Taken together these results demonstrate that the DvSnf7 dsRNA from a CRW-protected GM maize is unlikely to persist or accumulate in the soil environment. In a broader context, these results provide fundamental information to define environmental exposure scenarios for ecological risk assessment of future RNA-based agricultural products.
